# Facts and Misconceptions about 2D:4D, Social and Risk Preferences

**DOI:** 10.3389/fnbeh.2018.00022

**Published:** 2018-02-13

**Authors:** Judit Alonso, Roberto Di Paolo, Giovanni Ponti, Marcello Sartarelli

**Affiliations:** ^1^Departamento de Fundamentos de Análisis Económico, Universidad de Alicante, San Vicente del Raspeig/Sant Vicent del Raspeig, Alicante, Spain; ^2^Department of Economics, The University of Chicago, Chicago, IL, United States; ^3^Dipartimento di Economia e Finanza, Libera Università Internazionale degli Studi Sociali Guido Carli (LUISS), Rome, Italy

**Keywords:** 2D:4D, cognitive reflection, gender, risk, social preferences, C91, C92, D8

## Abstract

We study how the ratio between the length of the second and fourth digit (2D:4D) correlates with choices in social and risk preferences elicitation tasks by building a large dataset from five experimental projects with more than 800 subjects. Our results confirm the recent literature that downplays the link between 2D:4D and many domains of economic interest, such as social and risk preferences. As for the former, we find that social preferences are significantly lower when 2D:4D is above the median value only for subjects with low cognitive ability. As for the latter, we find that a high 2D:4D is not correlated with the frequency of subjects' risky choices.

## 1. Introduction

Research both in the hard sciences (e.g., Neurology and Physiology) and in the social sciences (e.g., Economics and Psychology) has increasingly focused on biological markers to improve our understanding of the biological basis of social behavior. Earlier research had claimed that prenatal exposure to sexual hormones has an effect on brain development that, in turn, influences individuals' decision making routines later in life (see for a survey Manning, 2002). Motivated by this evidence, a growing number of experimental studies has tested the relationship between the ratio between the second and fourth hand digit (2D:4D hereafter) -a marker which has been claimed to be negatively related to prenatal exposure to testosterone- and behavior in a wide variety of cognitive domains, including social and risk preferences.

Social preferences are a ubiquitous phenomenon in everyday life and have gained increasing attention in the social sciences. While there is robust evidence that shows that females exhibit more pronounced social concerns, only few studies have looked at their relationship with 2D:4D. Within this small set, Millet and Dewitte (2006) find a negative relationship between 2D:4D and giving in the dictator game. Using a variety of games, such as public good and dictator, Buser (2012) finds, instead, a positive relationship with giving. In related studies using the ultimatum game, Brañas-Garza et al. (2013) find that the relationship with giving follows an inverted U-shape while Van den Bergh and Dewitte (2006) find a negative relationship with rejection rates.

The relationship between 2D:4D and risk-taking has been widely studied experimentally to quantify the role played by innate traits in this type of decisions. Again, the evidence so far is mixed, as some studies find a negative relationship with the frequency of risky choices (e.g., Garbarino et al., 2011; Brañas-Garza et al., 2018) while others do not find any significant correlation (e.g., Apicella et al., 2008; Sapienza et al., 2009).

We contribute to this literature by assembling a meta-dataset consisting of five experimental projects involving 879 subjects in total. With this large dataset collecting evidence on behavioral tasks of a different nature, we first assess the relationship between 2D:4D and *inequity aversion* (Fehr and Schmidt, 1999), a proxy for social preferences that identifies the role of “envy” (i.e., *negative inequity aversion*) in comparison with “guilt” (i.e., *positive inequity aversion*). Second, we assess the relationship between 2D:4D and risk attitudes, which were elicited using Multiple Price Lists (Holt and Laury, 2002). Finally, following some recent contributions (Brañas-Garza et al., 2015; Cueva et al., 2016), we also assess the mediating role played by cognitive ability in the relationship between 2D:4D and subjects' decisions in both risk and distributional tasks.

We briefly summarize here our main results, that have been obtained by defining right hand 2D:4D high if it is greater than the gender-specific median value. When we look at social preferences, we find that for subjects with high 2D:4D the relationship with guilt is negative but not significant, whereas the relationship with envy is only significant and negative for subjects with low cognitive ability. If we, instead, use directly 2D:4D measures we find no significant association with social preferences. When we look at risk preferences, we find that the association between high 2D:4D and the frequency of risky choices is negative but not significant, with similar results holding if we use the raw 2D:4D index as a covariate. Overall, our empirical findings cannot but confirm some recent literature (discussed in section 2) which downplays the link between 2D:4D and behavior in experimental domains of interests, such as social and risk preferences.

The remainder of the paper is structured as follows. Section 2 reviews the related literature while section 3 describes the layout of our meta-dataset. In section 4, we report correlations between 2D:4D, gender and cognitive ability distilled from the debriefing questionnaire. In section 5 we report our findings on the relationship between 2D:4D and inequity aversion and in section 6 we look at risk attitudes. Finally, section 7 discusses our results and concludes, followed by an appendix collecting additional statistical evidence.

## 2. Literature review

The ratio between the length of the second (“index” finger) and fourth (“ring” finger) digit, also called second-to-fourth digit ratio (2D:4D), has been claimed to be a proxy for prenatal exposure to testosterone, with a lower ratio indicating higher exposure both for children and for adults (Manning et al., 1998). Related studies find a positive correlation between sex hormones at birth and 2D:4D measured at age 2 (Lutchmaya et al., 2004; Ventura et al., 2013). More recently Hollier et al. (2015) have challenged this view by providing evidence that the relationship between a measure of exposure to testosterone obtained using umbilical cord blood and 2D:4D measured at age 19-22 is not significant^1^. However, this result may be due by the fact that testosterone peaks between 12 and 18 weeks of gestation and decreases thereafter (Xie et al., 2017). In addition, in a replication study, (Hönekopp et al., 2007) find no systematic evidence of a relationship between 2D:4D and circulating sex hormones in adults. On the one hand, this result suggests that estimating the relationship between 2D:4D and proxies for decision-making without accounting for circulating testosterone does not lead to omitted variable bias. On the other, it suggests that additional research is awaited to obtain conclusive evidence on the relationship between 2D:4D and testosterone subjects are exposed to from gestation to adulthood.

Several studies have also shown that 2D:4D is a sexually dimorphic measure with, on average, males having lower 2D:4D than females (Putz et al., 2004). Moreover, earlier studies have reported that 2D:4D varies not only by gender, but also by ethnicity (Manning, 2002). It has also been found that these differences emerge prenatally and are stable during the developing years (Trivers et al., 2006). Voracek et al. (2007) carry out a wide replication study of published results on the relationship between 2D:4D and a variety of outcomes and, overall, confirm the results.

The literature on the relationship between 2D:4D and social preferences is scant and, again, results are mixed. Buser (2012) finds that in public good, dictator, trust and ultimatum games subjects with higher 2D:4D are more generous. By contrast, Brañas-Garza and Kovárík (2013) argue that, since 2D:4D measures in Buser (2012) are self-reported, his results may be affected by measurement error and biased if the error is correlated with one or more subjects' characteristics.

As for the experimental evidence on the dictator game, Millet and Dewitte (2006) find, instead, a negative relationship between 2D:4D and giving. In related experimental studies using ultimatum games, Van den Bergh and Dewitte (2006) find a negative relationship between 2D:4D and rejection rates while Brañas-Garza et al. (2013) find evidence of non-linearities in the relationship, with subjects with either high or low 2D:4D giving less. A non-linear relationship is also found by Sanchez-Pages and Turiegano (2010) for the one-shot prisoner's dilemma, with men with intermediate 2D:4D being more likely to cooperate^2^.

As for the relationship between 2D:4D and risk-taking behavior, results are mixed (see for a survey Apicella et al., 2015). Dreber and Hoffman (2007); Garbarino et al. (2011); Brañas-Garza et al. (2018) find a negative relationship for both genders, with Brañas-Garza et al. (2018) also finding that the relationship with a self-assessed and subjective measure of risk attitudes is not significant. Similarly, Ronay and von Hippel (2010); Brañas-Garza and Rustichini (2011); Stenstrom et al. (2011) find a negative relationship although only for males, with Brañas-Garza and Rustichini (2011) also finding that this result is mediated by a negative relationship between 2D:4D and abstract reasoning ability, an aspect of cognitive ability that was measured using the Raven Progressive Matrices task. In contrast, a number of studies find that the relationship is not significant at any conventional level (Apicella et al., 2008; Sapienza et al., 2009; Schipper, 2012; Aycinena et al., 2014; Drichoutis and Nayga, 2015)^3^.

## 3. Data and methods

We collect data from five experimental projects that were carried out at the Laboratory of Theoretical and Experimental Economics (LaTEx) of the Universidad de Alicante, from 2014 to 2017. The objects of these studies include, among others, risk and social preferences, which will be discussed in section 5 and 6 respectively. All experimental protocols are also endowed with a debriefing questionnaire from which we obtained information on subjects' gender and cognitive ability. Table 1 lists the projects in our meta-dataset and summarizes their structure^4^.

**Table 1:** Summary of experimental projects in the meta-dataset.

**Project**	**Reference**	**N**	**Topic**	**Social preferences**	**Risk preferences**	**2D:4D**
1	Albarran et al., 2017	279	Risk and uncertainty	No	Yes (89)	Yes
2	Cueva et al., 2016	96	Behavioral finance	No	Yes	Yes
3	Ponti *et al*., 2014	288	Entrepreneurship	Yes	Yes (96)	Yes
4	Ponti et al., 2017	144	Agency	Yes	Yes	Yes
5	Zhukova, 2017	72	Investment	No	Yes	Yes
		879		432	497	879

### 3.1. Behavioral evidence

The behavioral content of the five projects is as follows. Social preferences are elicited in projects 3 and 4 (432 subjects) and risk preferences are elicited in projects 1–5 (497 subjects).

#### 3.1.1. Social preferences

**Figure 1:**
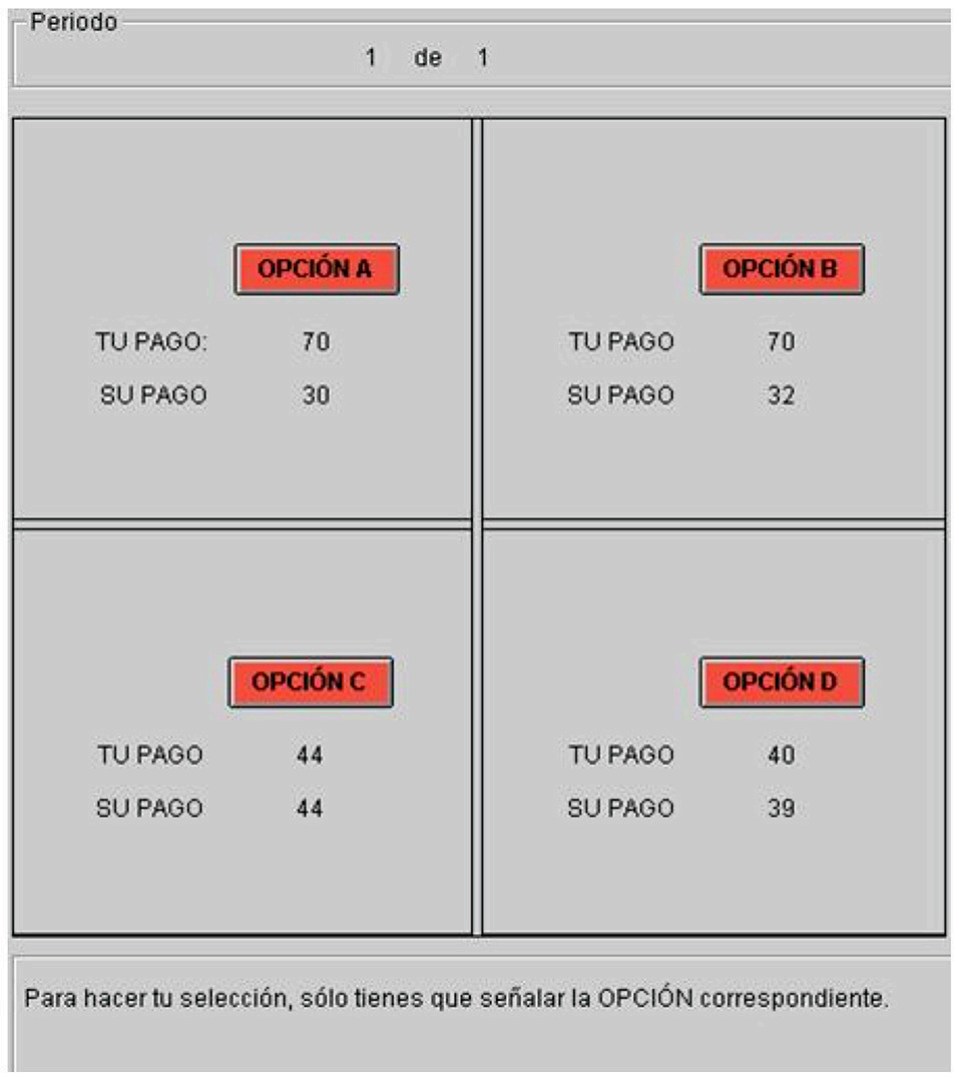
User interface for distributional decisions in projects 3 and 4.

As for social preferences, the elicitation protocol consists in a sequence of 24 distributional decisions, whose basic layout is borrowed from Cabrales et al. (2010). Subjects are matched in pairs and must choose one out of four options, as shown in Figure 1. An option corresponds to a pair of monetary prizes, one for each subject within the pair. At the beginning of each round *t* = 1, …, 24, subjects are informed about the option set $C_{t}=\left\{b^{k}\right\},k=1,\ldots ,4$. Each option $b^{k}=\left(b_{1}^{k},b_{2}^{k}\right)$ assigns a monetary prize, $b_{i}^{k}$, to player *i* = 1, 2, with $b_{1}^{k}\geq b_{2}^{k}$ for all *k*. In other words, player 1 (player 2) looks at the distributive problem associated with the choice of a specific option *k* from the viewpoint of the advantaged (disadvantaged) player, respectively.

Once choices are made, a “Random Dictator” protocol (Harrison and McDaniel, 2008) determines the payoff relevant decision, that is, an i.i.d. draw fixes the identity of the subject whose choice determines the monetary rewards for that pair and round. This design feature is particularly efficient when estimating inequity aversion in that, for roughly half of the observations we can identify separately, within-subject, individuals' attitudes toward *envy* (i.e., social preferences from a disadvantageous position) and *guilt* (i.e., social preferences from an advantageous position), respectively. After subjects have selected their favorite options, all payoff relevant information is revealed, and round payoffs are distributed.

#### 3.1.2. Risk preferences

**Figure 2:**
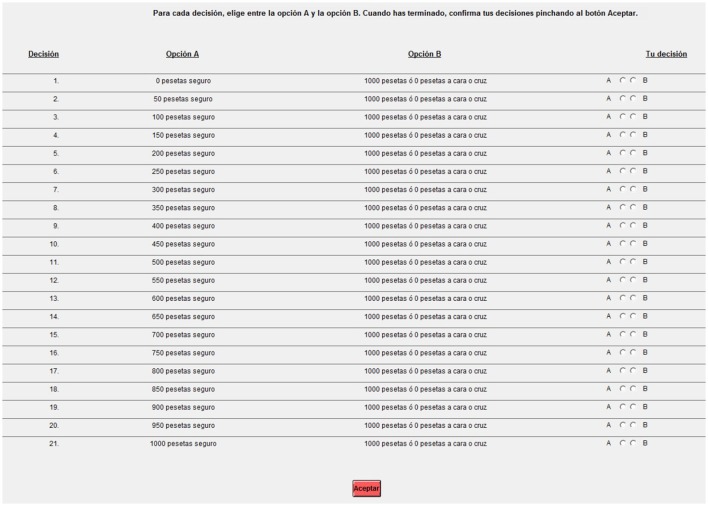
User interface for the multiple price list in projects 2–5.

Risk preferences have been elicited with a Multiple Price List (MPL, Holt and Laury, 2002) protocol in all projects, for a total of 497 subjects. In projects 2–5 our MPL protocol consists of a sequence of 21 binary choices. As Figure 2 shows, “Option A” corresponds to a sure payment whose value increases along the sequence from 0 to 1000 pesetas in steps of 50 while “Option B” is constant along the sequence and corresponds to a 50/50 chance to win 1,000 pesetas. In project 1, instead, the list consists of 16 binary choices: “Option A” is increasing from 0 to 15 euros in steps of 1 while “Option B” is a fixed lottery over three prizes drawn from Hey and Orme (1994). Subjects are asked to elicit their certain equivalent for 50 such lotteries. In both protocols one of the binary choices is selected randomly for payment at the end of the experiment^5^.

### 3.2. Individual characteristics

**Figure 3:**
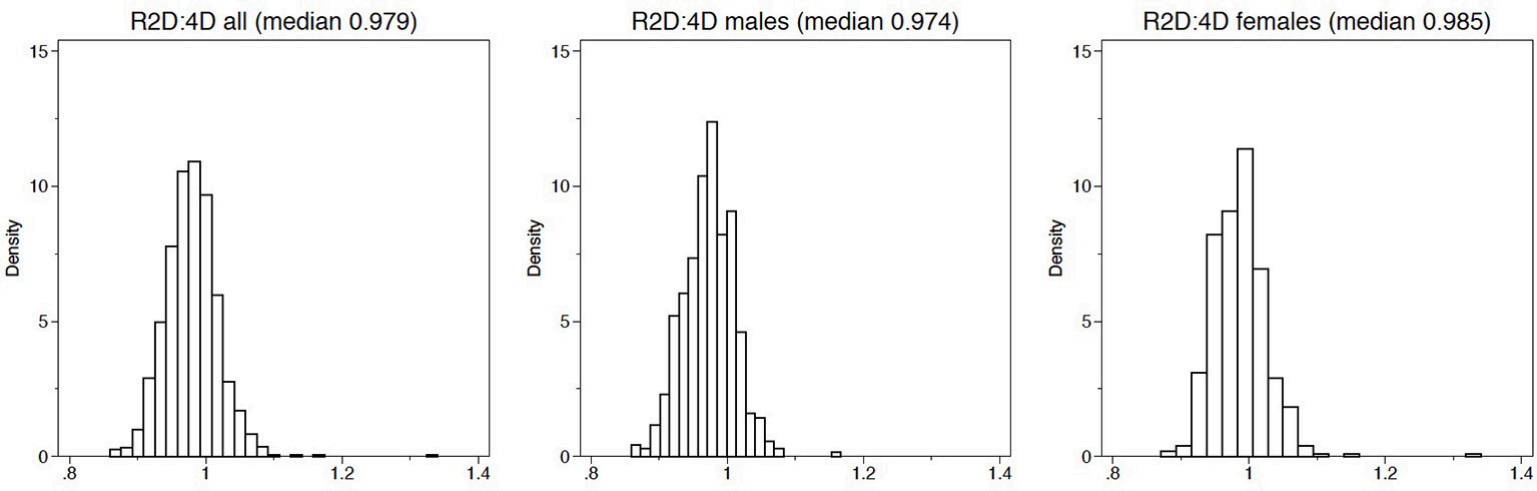
2D:4D histograms.

In all studies, we scanned both hands and we measured 2D:4D following the protocol set up by Neyse and Brañas-Garza (2014). By using this procedure, we avoid measurement errors usually associated with self-reported statements (Brañas-Garza and Kovárík, 2013). The 2D:4D measure reported in what follows is a dummy equal to 1 for subjects with a right hand 2D:4D above the gender-specific median value, high 2D:4D hereafter, and equal to 0 otherwise. This choice is based on the non-linear relationship between 2D:4D and behavioral outcomes that is reported in Brañas-Garza et al. (2013) among others. Gender difference in 2D:4D, with men exhibiting a lower 2D:4D as shown in Figure 3, have been taken into account by defining our binary measure of high or low 2D:4D by computing median values separately by gender. An additional advantage of using a dummy to discriminate between high and low 2D:4D rather than 2D:4D, that takes values in a very small interval around 1, is that it tends to simplify the interpretation of coefficients of interactions between the high 2D:4D dummy and other covariates in regressions^6^.

The Cognitive Reflection Test (CRT hereafter, Frederick, 2005) was administered in our debriefing questionnaire. It is a simple test of a quantitative nature especially designed to elicit the “predominant cognitive system at work” in respondents' reasoning:

CRT1. A bat and a ball cost 1.10 dollars. The bat costs 1.00 dollars more than the ball. How much does the ball cost? (Correct answer: 5 cents).
CRT2. If it takes 5 machines 5 min to make 5 widgets, how long would it take 100 machines to make 100 widgets? (Correct answer: 5 min).
CRT3. In a lake, there is a patch of lily pads. Every day, the patch doubles in size. If it takes 48 days for the patch to cover the entire lake, how long would it take for the patch to cover half of the lake? (Correct answer: 47 days).

The CRT provides not only a measure of cognitive ability, but also of *impulsiveness* and, possibly, other individuals' unobservable characteristics. In this test, the “impulsive” answer (10, 100, and 24, respectively) is shown to be the modal answer (Frederick, 2005). These answers, although incorrect, may have been selected by those subjects who do not think carefully enough. Following Cueva et al. (2016), we partition individuals into three groups. *Impulsive* subjects answer the erroneous intuitive value at least in two questions, *reflective* ones answer correctly at least two questions, and *others* are the residual group.

## 4. Results I: descriptive statistics

In this section we report descriptive statistics of 2D:4D and estimates of its correlation with the CRT score and with CRT categories dummies, our proxies for cognitive ability by way of pairwise correlations.

Figure 3 reports the distribution of 2D:4D in our meta-dataset for the full sample and separately for subsamples by gender. The distribution tends to be symmetric and the median value is slightly smaller than one for the full sample as well as for subsamples by gender. In addition, Figure 3 shows that 2D:4D tends to be smaller for males, in line with evidence that 2D:4D is sexually dymorphic in related studies.

Table 2 shows the correlations between 2D:4D, gender and proxies of cognitive ability. In addition, it report correlations using as a measure of prenatal exposure to testosterone a dummy equal to 1 if 2D:4D is greater than the gender-specific median and, also, a dummy equal to 1 if 2D:4D is either in the top or in the bottom tercile of the 2D:4D distribution by gender. The correlation between 2D:4D and the female dummy is positive and highly significant for both hands. 2D:4D is, instead, negatively and highly significantly correlated with the CRT reflective group dummy for the left hand when using the top-bottom tercile dummy. In addition, Table 2 shows that correlations between 2D:4D and the frequency of risky choices, our proxy for risk attitudes, are negative and, hence, qualitatively in line with results in related studies. However, estimates are not significant, even when using binary measures of prenatal exposure to testosterone. Since our proxies for social preferences are estimated parameters of Fehr and Schmidt (1999) model, the estimation procedure and their relationship with prenatal exposure to testosterone are reported in section 5^7^^,^^8^.

**Table 2:** Correlations.

	**2D:4D in level**		**Above median dummy**		**Top-bottom tercile dummy**	
	**L2D:4D**	**R2D:4D**	**LH2D:4D**	**HR2D:4D**	**TBL2D:4D**	**TBR2D:4D**
L2D:4D	1.000	0.628^***^	1.000	0.456^***^	1.000	0.185^***^
Female	0.177^***^	0.208^***^	−0.001	−0.001	−0.026	0.005
CRT	−0.066^**^	−0.049	−0.015	−0.002	−0.073^**^	−0.023
CRT Impulsive	0.047	0.037	0.001	0.008	0.052	0.046
CRT Reflective	−0.068^**^	−0.068^**^	−0.036	−0.029	−0.092^***^	−0.026
CRT Other	0.014	0.027	0.039	0.022	0.035	−0.032
Freq. of risky choices	−0.043	−0.034	−0.004	−0.026	−0.011	0.029

** *p < 0.05*,

*** *p < 0.01*.

## 5. Results II: social preferences

This section frames Dictators' behavior in projects 3 and 4 within the realm of Fehr and Schmidt (1999), one of the most popular models of social preferences. According to it, the Dictator's utility associated to option *k*, *u*(*k*), does not only depend on the Dictator's own monetary payoff, $x_{D}^{k}$, but also on that of the Recipient, $x_{R}^{k}$, as follows:

(1) $$\begin{array}{r} u\left(k\right)=x_{D}^{k}-\alpha \text{max}\left[x_{R}^{k}-x_{D}^{k},0\right]-\beta \text{max}\left[x_{D}^{k}-x_{R}^{k},0\right], \end{array}$$

where the values of α and β determine the Dictator's *envy* (i.e., aversion to inequality when receiving less than the Recipient) and *guilt* (i.e., aversion to inequality when receiving more than the Recipient), respectively.

In what follows we shall estimate by maximum likelihood, for each participant, the two coefficients of Equation (1) by way of a standard multinomial logit model.

**Figure 4:**
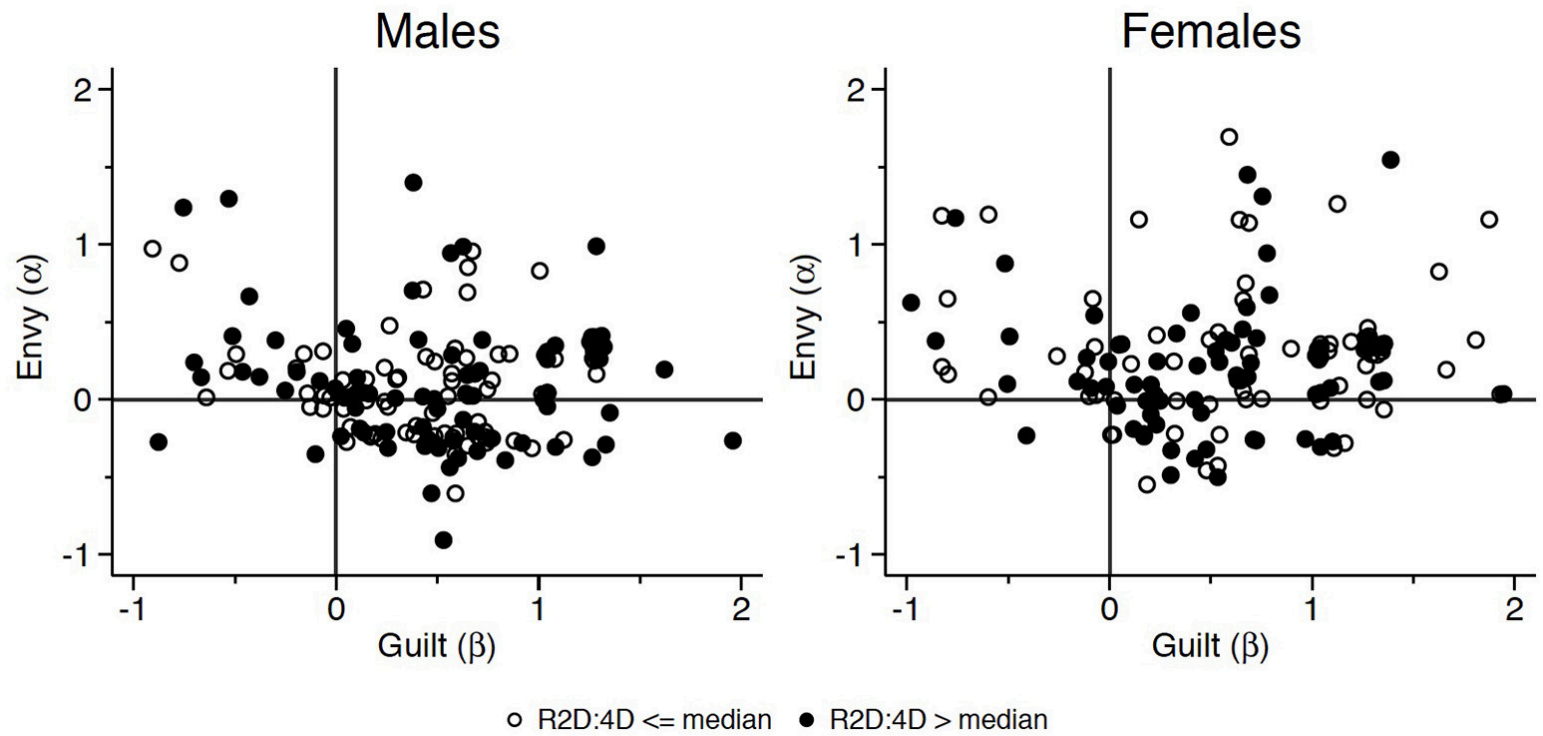
Social preferences: individual estimates.

Figure 4 reports the estimated coefficients of equation (1) for each subject participating in the experiment, disaggregated by gender and by whether the right hand 2D:4D is above the gender-specific median. By conditioning on the gender-specific median, we control for the correlation between gender and 2D:4D that we detected in Table 2. As Figure 4 shows, (*i*) estimates for males are less dispersed with respect to the origin (corresponding to more “selfish” preferences) and (*ii*) inequity aversion appears to be the modal distributional type, with specific reference to females with low 2D:4D. The pooled estimates of α and β for the full sample (clustered at the subject level) are 0.288 (std. err. 0.001, *p* = 0.000) and 0.684 (std. err. 0.008, *p* = 0.000), respectively^9^.

In order to quantify the relationship between 2D:4D and inequity aversion, we follow a semi-parametric approach. First, for both α and β, we partition our subject pool into three subsets, depending on whether the corresponding individual-level estimates are significantly smaller than zero (53 and 28 for α and β respectively), not significantly different (130 and 160), or significantly greater (159 and 154). We then set up an ordered probit regression by which the probability of falling in each category is a function of high 2D:4D dummy, gender and the CRT groups, with the reflective group as omitted category. Our choice of using a dummy equal to 1 if 2D:4D is above the gender-specific median, rather than 2D:4D itself, may be subject to problems, such as a lower statistical power and a higher probability of type I or II errors (Irwin and McClelland, 2003; McClelland et al., 2015). However, by using non-linear models to estimate the relationship between 2D:4D and social preferences in this section, our estimates are unlikely to suffer from such problems^10^.

Table 3 reports the estimated coefficients, with alternative sets of covariates being used. We start estimating the relationship between social preferences and the high 2D:4D dummy (HR2D:4D) in model (1) without adding any additional control and then, in model (2) and (3) we add female and CRT categories dummies to assess if they play a mediating role. In model (4) we use an interaction term between HR2D:4D and the female dummy to account for the positive correlation between gender and 2D:4D we observed in Table 2. Finally, in model (5) we use an interaction term between the CRT categories dummies and HR2D:4D. In addition, we report in Table 3 marginal effects (MFX) of HR2D:4D, evaluated at the sample mean, while MFX with respect to gender and CRT are shown in Appendix A (Supplementary Material)^11^.

**Table 3:** Ordered probit regressions of social preferences individual estimates.

	**(1)**		**(2)**		**(3)**		**(4)**		**(5)**	
	**α**	**β**	**α**	**β**	**α**	**β**	**α**	**β**	**α**	**β**
HR2D:4D (HR)	−0.064	−0.235^*^	−0.066	−0.236^*^	−0.068	−0.213^*^	0.018	−0.185	0.395	−0.562^**^
	(0.123)	(0.125)	(0.124)	(0.125)	(0.124)	(0.126)	(0.168)	(0.172)	(0.249)	(0.254)
Female (F)			0.376^***^	0.097	0.326^***^	0.062	0.423^**^	0.093	0.326^**^	0.069
			(0.124)	(0.125)	(0.126)	(0.127)	(0.180)	(0.181)	(0.127)	(0.127)
CRT Imp (CRTI)					0.359^**^	0.333^**^	0.355^**^	0.332^**^	0.604^***^	0.111
					(0.149)	(0.151)	(0.149)	(0.151)	(0.208)	(0.214)
CRT Others (CRTO)					0.269	−0.120	0.269	−0.121	1.034^***^	−0.441
					(0.215)	(0.214)	(0.215)	(0.214)	(0.351)	(0.330)
HR × F							−0.189	−0.060		
							(0.249)	(0.251)		
HR × CRTI									−0.494^*^	0.440
									(0.294)	(0.300)
HR × CRTO									−1.298^***^	0.579
									(0.449)	(0.433)
MFX P(α > 0) of HR	−0.025		−0.026		−0.027		−0.029		−0.034	
S.e.	0.049		0.049		0.049		0.049		0.050	
MFX P(β > 0) of HR		−0.093^*^		−0.093^*^		−0.084^*^		−0.084^*^		−0.082^*^
S.e.		0.049		0.049		0.050		0.050		0.050
N	342		342		342		342		342	

*Standard errors in parentheses*.

* *p < 0.10*,

** *p < 0.05*,

*** *p < 0.01*.

Table 3 shows that the relationship between HR2D:4D and negative inequity aversion, i.e., envy, is negative and the same holds for the relationship with positive inequity aversion, i.e., guilt. MFX, which are reported at the bottom of the table, show that the relationship with envy or with guilt is not significant. The table also shows that envy is higher for females while the impulsive group (CRTI) is characterized by higher envy and higher guilt than the reflective group, which is the excluded CRT category. These estimates are significant as shown by MFX in Appendix A (Supplementary Material). These results hold for the five econometric specifications reported in Table 3, as shown by MFX in Appendix A (Supplementary Material). Finally, when we interact the HR2D:4D dummy with CRT categories to assess if the influence of 2D:4D differs by subjects' cognition, we find that subjects with high 2D:4D and low cognitive ability, proxied by the CRT impulsive dummy, do not exhibit significantly lower envy than subjects with high 2D:4D in the CRT reflective group, while the relationship is significant when considering the CRT residual group dummy^12^^,^^13^.

## 6. Results III: risk attitudes

In this section we study the relationship between 2D:4D and proxies for risk preferences by using data on 497 subjects from all projects. Risk preferences are elicited by way of a Multiple Price List (MPL, Holt and Laury, 2002), in which individuals have to choose between two alternatives: a list of increasing sure payments and a lottery. Since the same protocol has been used in projects 2 to 5 while the number of decisions, lottery prizes, the experimental currency and their probability distribution differ in project 1, we choose two proxies for risk preferences that we believe are not affected by these differences.

Following Cueva et al. (2016), we define *consistent* those individuals whose decisions satisfy two conditions: (*i*) start by choosing the lottery option, as it stochastically dominates the sure payment of 0, and (*ii*) switch only once at some point along the price list to the sure payment and stick to it up to the end. We can use data from all projects in our empirical analysis as none of the differences between our MPL protocols has an impact on the consistency definition. We also define a dummy equal to 1 if the proportion of risky choices made by a subject, i.e., the ratio between the number of lotteries chosen in the list and the total number of decisions, is greater than the median value. By using the proportion rather than the number of risky choices, we control for the difference in the design of the MPL in project 1.

Table 4 shows linear probability estimates of subjects' consistency dummy. In addition to the high 2D:4D dummy, our covariates include dummies for females and for the CRT groups, as well as for the interaction between the high 2D:4D dummy, female and CRT groups dummies. The top panel of the table shows regression estimates while the bottom one marginal effects (MFX) for those specifications in which we used interaction terms, evaluated at the sample mean. Because of the differences in the experimental protocol of project 1 with respect to the others, we also include a dummy equal to 1 for subjects in project 1 in order to absorb project-specific effects.

**Table 4:** Subjects' consistency in risky choices.

	**(1)**	**(2)**	**(3)**	**(4)**	**(5)**
HR2D:4D	0.071^*^	0.072^*^	0.069^*^	0.047	−0.039
	(0.037)	(0.037)	(0.036)	(0.049)	(0.055)
Female (F)		−0.057	−0.025	−0.048	−0.023
		(0.037)	(0.038)	(0.056)	(0.038)
CRT Imp. (CRTI)			−0.164^***^	−0.163^***^	−0.240^***^
			(0.039)	(0.039)	(0.053)
CRT Other. (CRTO)			−0.152^***^	−0.152^***^	−0.202^***^
			(0.052)	(0.052)	(0.073)
HR2D:4D × F				0.047	
				(0.073)	
HR2D:4D × CRTI					0.149^**^
					(0.074)
HR2D:4D × CRTO					0.095
					(0.104)
Project 1	0.066	0.069	0.066	0.066	0.067
	(0.044)	(0.044)	(0.044)	(0.044)	(0.045)
Constant	0.737^***^	0.764^***^	0.879^***^	0.889^***^	0.934^***^
	(0.029)	(0.032)	(0.034)	(0.038)	(0.036)
MFX of F				−0.025	
S.e.				0.039	
MFX of CRTI					−0.166^***^
S.e.					0.039
MFX of CRTO					−0.155^***^
S.e.					0.052
MFX of HR				0.069^*^	0.069^*^
S.e.				0.037	0.036
N	497	497	497	497	497

*Robust standard errors in parentheses*.

* *p < 0.10*,

** *p < 0.05*,

*** *p < 0.01*.

When we look at estimates in Table 4, we find that the probability of being consistent in their decisions is higher for subjects with a high 2D:4D but the difference is not significant, that there is no significant gender difference and that it is significantly lower for subjects in the impulsive (CRTI) or in the residual (CRTO) group than for the reflective group. We see no changes when we include the interaction between female and the high 2D:4D variable, suggesting that they do not play any mediating role. When we add interaction terms between the high 2D:4D dummy and the female dummy, we find no significant gender differences in the relationship between 2D:4D and consistency. When we add interactions between high 2D:4D and cognitive ability dummies, the high 2D:4D dummy coefficient is no longer significant while the coefficient of the interaction with the CRTI dummy is positive and significant, suggesting that subjects in the CRT impulsive group and with high 2D:4D are more consistent. When looking at MFX, we find that consistency is significantly lower for subjects with low cognitive ability, it is higher for subjects with a high 2D:4D although the difference is not significant^14^.

Table 5 shows linear probability estimates for consistent subjects of a dummy equal to 1 if the proportion of risky choices is greater than the median. We find no significant relationship with the high 2D:4D dummy while the probability is significantly lower for females. Results are unchanged when using 2D:4D or the top-bottom tercile dummy, as shown in Appendix A (Supplementary Material)^15^^,^^16^^,^^17^.

**Table 5:** Consistent subjects' relative frequency of risky choices above median.

	**(1)**	**(2)**	**(3)**	**(4)**	**(5)**
HR2D:4D (HR)	0.005	0.006	0.006	−0.009	−0.041
	(0.017)	(0.017)	(0.017)	(0.020)	(0.031)
Female (F)		−0.058^***^	−0.056^***^	−0.073^***^	−0.056^***^
		(0.017)	(0.018)	(0.025)	(0.018)
CRT Imp. (CRTI)			−0.007	−0.006	−0.036
			(0.020)	(0.020)	(0.028)
CRT Other. (CRTO)			0.007	0.007	−0.030
			(0.025)	(0.025)	(0.037)
HR2D:4D × F				0.033	
				(0.034)	
HR2D:4D × CRTI					0.058
					(0.038)
HR2D:4D × CRTO					0.072
					(0.050)
Project 1	−0.064^***^	−0.058^***^	−0.058^***^	−0.057^***^	−0.056^***^
	(0.019)	(0.019)	(0.019)	(0.019)	(0.019)
Constant	0.453^***^	0.478^***^	0.480^***^	0.487^***^	0.503^***^
	(0.012)	(0.013)	(0.018)	(0.019)	(0.023)
MFX of F				−0.057^***^	
S.e.				0.018	
MFX of CRTI					−0.007
S.e.					0.020
MFX of CRTO					0.006
S.e.					0.025
MFX of HR				0.007	0.008
S.e.				0.017	0.017
N	390	390	390	390	390

*Robust standard errors in parentheses*.

*** *p < 0.01*.

## 7. Discussion

When we look at social preferences, we contribute to the literature that has almost entirely focused on giving as a proxy for social preferences in a variety of experimental settings (e.g., Buser, 2012; Brañas-Garza et al., 2013) by isolating two aspects underlying the incentives to give, that is, envy and guilt. Finding a negative and significant relationship between 2D:4D and envy, i.e., less generous behavior by subjects when they play in the disadvantaged role, only for subjects with low cognitive ability and non-significant results for guilt suggests that individual heterogeneity may play a role in reconciling the mixed evidence on the relationship between 2D:4D and giving in the literature. However, giving and inequity aversion are not fully comparable proxies for social preferences as they are used in different experimental settings.

Although evidence of heterogeneity by ability in the relationship between 2D:4D and subjects' decision-making has been documented in risky choices (Brañas-Garza and Rustichini, 2011), we are the first to do so in the realm of social preferences, to the best of our knowledge. Finding that subjects with high 2D:4D and low cognitive ability exhibit significantly lower envy than subjects with low 2D:4D and high cognitive ability shows evidence of heterogeneity by ability in the relationship between social preferences and 2D:4D. This result, by suggesting an attenuating role of low cognitive ability and high 2D:4D on inequity aversion contributes to related studies, for example Cueva et al. (2016) and Ponti and Rodriguez-Lara (2015), who find that the CRT impulsive category exhibits higher inequity aversion.

When we look at risk attitudes, we find that the relationship between 2D:4D and the probability that the number of risky decisions is above the median, shows a mixed sign, it is quantitatively small and never significant. These results contribute to the related literature as the sign and significance of the relationship is not conclusive. Overall, this may be due to the fact that there is genuinely no relationship between 2D:4D and risky decisions or, alternatively, to differences across studies. The composition of the subject pool may play a role if the willingness to participate in an experiment correlates with subjects' socio-economic background and risk aversion. In addition, the type of risk preferences elicitation task may also matter. For example, studies that, including ours, use a task in which subjects can choose a risk-free option tend to find a non-significant association while studies in which subjects choose between two lotteries tend to find a negative and significant association.

After discussing our results relative to those in related studies, we now critically assess them in the light of potential methodological issues, that we believe all researchers wanting to contribute to this interdisciplinary literature should bear in mind. Studies in hard sciences of the relationship between direct measures of prenatal exposure to testosterone and 2D:4D find mixed results, whose sign and significance seem to depend critically on whether direct measures are obtained in an early stage *in utero* or, instead, close to the birth. Studies in social sciences on the relationship between 2D:4D and decision-making find mixed results that may depend on the accuracy of 2D:4D measurement and, in addition, to the experimental tasks used to elicit subjects' preferences. Overall, this suggests both that additional research is awaited to reconcile existing differences across studies in the literature and that caution is used in the interpretation of results before these differences are better understood.

## Author contributions

All authors listed have made a substantial, direct and intellectual contribution to the work, and approved it for publication.

### Conflict of interest statement

The authors declare that the research was conducted in the absence of any commercial or financial relationships that could be construed as a potential conflict of interest.

## Footnotes

^1^See Kaltwasser et al. (2017) for analogous findings.

^2^Related studies manipulate experimentally hormones levels and estimate their relationship with proxies for social preferences. Zak et al. (2009) increase the level of circulating testosterone and find that it decreases giving in ultimatum games. Kosfeld et al. (2005); Zak et al. (2007) increase, instead, levels of oxytocin, a hormone that is hypothesized to increase empathy in humans, and find that it has a positive impact on giving in ultimatum games but not in dictator games, which they interpret as evidence of generosity. In addition, neuroeconomic evidence shows that exposure to prenatal hormones (testosterone or estrogen) may affect the activity in specific brain areas that are associated with individuals' behavior in several settings and with their personality (Fehr and Camerer, 2007; Lee, 2008; Fehr and Krajbich, 2009).

^3^In a non-experimental setting Coates et al. (2009) find a negative relationship between 2D:4D, profitability and tenure on the job for a sample of 49 financial traders in the City of London. In a related although different experimental setting that involves strategic interactions among subjects, Pearson and Schipper (2012) find no significant association between 2D:4D, bids in sealed bid first-price auctions and subjects' total payoffs. A positive relationship is also found between 2D:4D, risky choices and criminality using field data, although with a low number of observations in Hanoch et al. (2012).

^4^Approval for the experiment was given by the LaTEx Ethics Committee. Participants gave their consent to participate in social experiments when they signed up in ORSEE (Greiner, 2004), the online recruitment tool used at LaTEx. When, before the experiment started, instructions about its content were read aloud to all participants, they were informed that they could leave the experiment at any stage. Separate approvals were obtained for each of the five experimental studies used in the paper.

^5^The interested reader in the estimation of risk preferences in a setting with several identical rounds, in which subjects may learn over rounds, can refer to Albarran et al. (2017).

^6^In section 5 we discuss the advantages and disadvantages of using the high 2D:4D dummy rather than 2D:4D itself. For the sake of robustness, we also report results of our analysis with 2D:4D in Appendix A (Supplementary Material).

^7^The interested reader can find additional statistical evidence on the relationship between 2D:4D and personality traits in Alonso et al. (2017), the working paper version of this manuscript.

^8^Out of our 879 subjects CRT reflective, with 2 or more correct answers are 149 (16.7%), CRT impulsive, with at least one incorrect and impulsive answers, are 531 (60.4%) and the residual group contains 199 (22.6%).

^9^These figures are consistent with previous results (take, e.g., Cabrales et al., 2010).

^10^We also set up a bivariate ordered probit estimation in which we allow error terms in the equations of α and β to be jointly distributed. We find that the covariance parameter is not significant.

^11^The number of observations shown at the bottom of Table 3 is lower than the total number of subjects in projects 3 and 4 since we dropped those subjects for whom maximum likelihood estimation of α and β did not converge.

^12^Marginal effects are the same when we estimate them using, as an alternative measure, 2D:4D in levels, except the estimated relationship with guilt.

^13^When we replicated our main experimental results by using a dummy equal to 1 if 2D:4D is either in the bottom tercile of the distribution or in the top one, as a sensitivity analysis, we obtained similar results, except a positive and significant relationship between envy and the top-bottom tercile dummy, as shown in Appendix A (Supplementary Material). Most of the results shown in this section on the relationship between 2D:4D and social preferences tend to lose significance when they are obtained with the high 2D:4D dummy defined using left hand 2D:4D, as shown in Appendix A (Supplementary Material).

^14^Estimates of the same regression except for using, rather than the high 2D:4D dummy, 2D:4D itself or the top-bottom tercile dummy are reported in Appendix A (Supplementary Material). We can see some differences depending on the measure used: the probability of consistency is lower for females when we use 2D:4D and also when we use the top-bottom tercile dummy, although the estimates are not significant.

^15^Estimates of Table 5 obtained using the full sample are not reported as they are in line with those obtained using only observations of consistent subjects.

^16^Results are qualitatively unchanged when using a logit model or when the dummy equal to 1 if the frequency of risky choices is above the median, one of the dependent variables, is defined using median values separately for projects 1 since the certain equivalent is different from projects 2 to 5. They are not reported although they are available upon request. As a sensitivity analysis, we replicated our main experimental results by using 2D:4D and a dummy equal to 1 if 2D:4D is either in the bottom tercile of the distribution or in the top one and obtained similar results and obtain similar results. This seems to suggest that, at least in our case, estimates of regressions using the high 2D:4D dummy are not severely biased, as suggested by Irwin and McClelland (2003); McClelland et al. (2015).

^17^Most of the results shown in this section on the relationship between 2D:4D and risk attitudes do not hold when they are obtained with the high 2D:4D dummy defined using left hand 2D:4D, as shown in Appendix A (Supplementary Material).
