# Supplementary material for: Facts and Misconceptions about 2D:4D, Social and Risk Preferences
Source: Front Behav Neurosci. 2018 Feb 13;12:22. doi: 10.3389/fnbeh.2018.00022 (PMC5816919; doi:10.3389/fnbeh.2018.00022)
Supplement: Supplementary file 1 [file DataSheet1.pdf]

Some (Mis)facts about 2D:4D, Social and Risk Preferences. *Online Appendix**

Judit Alonso
Universidad de Alicante

Roberto Di Paolo
Universidad de Alicante

Giovanni Ponti
Universidad de Alicante
The University of Chicago
LUISS Guido Carli Roma

Marcello Sartarelli
Universidad de Alicante†

February 6, 2018

Abstract

This Appendix shows additional statistical evidence on the relationship between 2D:4D, social and risk preferences that offers support to the validity of our main results.

Keywords: 2D:4D, cognitive reflection, gender, risk, social preferences
JEL Classification: C91, C92, D8

*Financial support from the Spanish Ministerio de Economía y Competitividad (ECO2013-43119, ECO2015-65820-P and ECO2016-77200-P), Universidad de Alicante (GRE 13-04), Generalitat Valenciana (Research Projects Grupos 3/086) and Instituto Valenciano de Investigaciones Económicas (IVIE) is gratefully acknowledged.

†Corresponding author. Departamento de Fundamentos del Análisis Económico, Universidad de Alicante, Apartado de Correos 99, 03080 Alicante. E-mail: marcellosartarelli@gmail.com

Table 1: Ordered probit regressions of social preferences individual estimates and marginal effects by using a dummy = 1 when the Right 2D:4D ratio (R2D:4D) higher than the gender specific median.

	(1)		(2)		(3)		(4)		(5)	
	α	β	α	β	α	β	α	β	α	β
HR2D:4D (HR)	-0.064	-0.235*	-0.066	-0.236*	-0.068	-0.213*	0.018	-0.185	0.395	-0.562**
	(0.123)	(0.125)	(0.124)	(0.125)	(0.124)	(0.126)	(0.168)	(0.172)	(0.249)	(0.254)
Female (F)			0.376***	0.097	0.326***	0.062	0.423**	0.093	0.326**	0.069
			(0.124)	(0.125)	(0.126)	(0.127)	(0.180)	(0.181)	(0.127)	(0.127)
CRT Imp (CRTI)					0.359**	0.333**	0.355**	0.332**	0.604***	0.111
					(0.149)	(0.151)	(0.149)	(0.151)	(0.208)	(0.214)
CRT Others (CRTO)					0.269	-0.120	0.269	-0.121	1.034***	-0.441
					(0.215)	(0.214)	(0.215)	(0.214)	(0.351)	(0.330)
HR × F							-0.189	-0.060		
							(0.249)	(0.251)		
HR × CRTI									-0.494*	0.440
									(0.294)	(0.300)
HR × CRTO									-1.298***	0.579
									(0.449)	(0.433)
MFX P(α=0) of HR	0.010		0.011		0.011		0.012		0.015	
S.e.	0.020		0.020		0.021		0.021		0.022	
MFX P($\alpha > 0$) of HR	-0.025		-0.026		-0.027		-0.029		-0.034	
S.e.	0.049		0.049		0.049		0.049		0.050	
MFX P(α=0) of F			-0.062***		-0.054**		-0.055**		-0.057**	
S.e.			0.022		0.022		0.023		0.023	
MFX P($\alpha > 0$) of F			0.149***		0.129***		0.130***		0.129***	
S.e.			0.049		0.050		0.050		0.050	
MFX P($\alpha = 0$) of CRTI					-0.054**		-0.054**		-0.056**	
S.e.					0.022		0.022		0.023	
MFX P($\alpha > 0$) of CRTI					0.141**		0.140**		0.140**	
S.e.					0.058		0.058		0.058	
MFX P($\alpha = 0$) of CRTO					-0.051		-0.051		-0.079	
S.e.					0.046		0.046		0.053	
MFX P($\alpha > 0$) of CRTO					0.107		0.107		0.152	
S.e.					0.085		0.085		0.087	
MFX P(β=0) of HR		0.058*		0.058*		0.053*		0.053*		0.052
S.e.		0.031		0.031		0.032		0.032		0.032
MFX P($\beta > 0$) of HR		-0.093*		-0.093*		-0.084*		-0.084*		-0.082*
S.e.		0.049		0.049		0.050		0.050		0.050
MFX P(β=0) of F				-0.024		-0.016		-0.016		-0.017
S.e.				0.031		0.032		0.032		0.032
MFX P($\beta > 0$) of F				0.038		0.025		0.025		0.027
S.e.				0.049		0.050		0.050		0.050
MFX P($\beta = 0$) of CRTI						-0.079**		-0.079**		-0.079**
S.e.						0.035		0.035		0.035
MFX P($\beta > 0$) of CRTI						0.130**		0.130**		0.129**
S.e.						0.058		0.058		0.058
MFX P($\beta = 0$) of CRTO						0.029		0.029		0.036
S.e.						0.049		0.048		0.048
MFX P($\beta > 0$) of CRTO						-0.047		-0.048		-0.059
S.e.						0.083		0.083		0.084
N	342		342		342		342		342	

Standard errors in parentheses. * $p < 0.10$, ** $p < 0.05$, *** $p < 0.01$

Table 2: Ordered probit regressions of social preferences individual estimates and marginal effects with R2D:4D in levels

	(1)		(2)		(3)		(4)		(5)	
	α	β	α	β	α	β	α	β	α	β
HR2D:4D (HR)	-0.097	-0.683	-1.237	-1.007	-1.426	-0.819	-1.746	-1.138	7.488**	-6.244*
	(1.638)	(1.656)	(1.688)	(1.698)	(1.700)	(1.711)	(2.234)	(2.266)	(3.513)	(3.526)
Female (F)			0.396***	0.112	0.348***	0.074	-0.395	-0.652	0.348***	0.082
			(0.128)	(0.128)	(0.129)	(0.130)	(3.366)	(3.381)	(0.130)	(0.130)
CRT Imp (CRTI)					0.364**	0.339**	0.365**	0.340**	10.304***	-5.576
					(0.149)	(0.151)	(0.149)	(0.151)	(3.957)	(3.962)
CRT Others (CRTO)					0.279	-0.134	0.280	-0.133	20.628***	-12.756**
					(0.216)	(0.214)	(0.216)	(0.214)	(6.234)	(6.136)
HR × F							0.757	0.740		
							(3.428)	(3.446)		
HR × CRTI									-10.208**	6.072
									(4.058)	(4.060)
HR × CRTO									-20.719***	12.859**
									(6.328)	(6.239)
MFX P($\alpha = 0$) of HR	0.015		0.202		0.237		0.229		0.295	
S.e.	0.261		0.276		0.283		0.283		0.307	
MFX P($\alpha > 0$) of HR	-0.039		-0.491		-0.566		-0.550		-0.657	
S.e.	0.651		0.669		0.673		0.677		0.682	
MFX P($\alpha = 0$) of F			-0.065***		-0.058**		-0.058**		-0.062**	
S.e.			0.023		0.023		0.023		0.025	
MFX P($\alpha > 0$) of F			0.156***		0.138***		0.137***		0.138***	
S.e.			0.050		0.051		0.051		0.051	
MFX P($\alpha = 0$) of CRTI					-0.055**		-0.055***		-0.052**	
S.e.					0.022		0.021		0.024	
MFX P($\alpha > 0$) of CRTI					0.143**		0.144**		0.125**	
S.e.					0.058		0.058		0.059	
MFX P($\alpha = 0$) of CRTO					-0.053		-0.053		-0.074	
S.e.					0.046		0.046		0.053	
MFX P($\alpha > 0$) of CRTO					0.111		0.111		0.141	
S.e.					0.085		0.085		0.088	
MFX P($\beta = 0$) of HR		0.167		0.246		0.205		0.196		0.183
S.e.		0.405		0.415		0.427		0.428		0.427
MFX P($\beta > 0$) of HR		-0.270		-0.398		-0.324		-0.311		-0.291
S.e.		0.655		0.671		0.676		0.679		0.679
MFX P($\beta = 0$) of F				-0.027		-0.019		-0.018		-0.020
S.e.				0.032		0.033		0.033		0.033
MFX P($\beta > 0$) of F				0.044		0.029		0.029		0.032
S.e.				0.051		0.051		0.051		0.051
MFX P($\beta = 0$) of CRTI						-0.080*		-0.080*		-0.085*
S.e.						0.034		0.034		0.034
MFX P($\beta > 0$) of CRTI						0.132**		0.133**		0.142**
S.e.						0.058		0.058		0.058
MFX P($\beta = 0$) of CRTO						0.032		0.031		0.040
S.e.						0.048		0.047		0.046
MFX P($\beta > 0$) of CRTO						-0.053		-0.052		-0.068
S.e.						0.083		0.083		0.084
N	342		342		342		342		342	

Standard errors in parentheses. * $p < 0.10$, ** $p < 0.05$, *** $p < 0.01$

Table 3: Ordered probit regressions of social preferences individual estimates and marginal effects by using a dummy = 1 if R2D:4D in the top-bottom tercile

	(1)		(2)		(3)		(4)		(5)	
	α	β	α	β	α	β	α	β	α	β
HR2D:4D (HR)	0.018	-0.102	0.013	-0.103	0.017	-0.100	0.128	-0.121	0.331	0.198
	(0.130)	(0.132)	(0.131)	(0.132)	(0.131)	(0.133)	(0.176)	(0.181)	(0.263)	(0.265)
Female (F)			0.375***	0.096	0.326**	0.062	0.490**	0.032	0.326***	0.065
			(0.124)	(0.125)	(0.126)	(0.127)	(0.216)	(0.218)	(0.127)	(0.127)
CRT Imp (CRTI)					0.359**	0.337**	0.360**	0.336**	0.652**	0.616**
					(0.149)	(0.151)	(0.149)	(0.151)	(0.255)	(0.258)
CRT Others (CRTO)					0.260	-0.141	0.269	-0.143	0.464	0.049
					(0.215)	(0.213)	(0.215)	(0.214)	(0.376)	(0.371)
HR × F							-0.247	0.045		
							(0.264)	(0.266)		
HR × CRTI									-0.440	-0.422
									(0.311)	(0.315)
HR × CRTO									-0.305	-0.289
									(0.457)	(0.453)
MFX P($\alpha = 0$) of HR	-0.003		-0.002		-0.003		-0.002		-0.002	
S.e.	0.020		0.021		0.022		0.022		0.022	
MFX P($\alpha > 0$) of HR	0.007		0.005		0.007		0.004		0.005	
S.e.	0.052		0.052		0.052		0.052		0.052	
MFX P($\alpha = 0$) of F			-0.061***		-0.054**		-0.055**		-0.055**	
S.e.			0.022		0.022		0.023		0.023	
MFX P($\alpha > 0$) of F			0.148***		0.129***		0.129***		0.129***	
S.e.			0.049		0.050		0.050		0.050	
MFX P($\alpha = 0$) of CRTI					-0.054**		-0.054**		-0.054**	
S.e.					0.022		0.022		0.022	
MFX P($\alpha > 0$) of CRTI					0.141**		0.141**		0.142**	
S.e.					0.058		0.058		0.058	
MFX P($\alpha = 0$) of CRTO					-0.049		-0.051		-0.050	
S.e.					0.045		0.046		0.046	
MFX P($\alpha > 0$) of CRTO					0.103		0.107		0.104	
S.e.					0.085		0.085		0.085	
MFX P($\beta = 0$) of HR		0.025		0.026		0.025		0.025		0.027
S.e.		0.033		0.033		0.034		0.034		0.035
MFX P($\beta > 0$) of HR		-0.040		-0.041		-0.040		-0.039		-0.042
S.e.		0.052		0.052		0.053		0.053		0.053
MFX P($\beta = 0$) of F				-0.024		-0.016		-0.016		-0.016
S.e.				0.031		0.032		0.032		0.032
MFX P($\beta > 0$) of F				0.038		0.025		0.025		0.026
S.e.				0.049		0.050		0.050		0.050
MFX P($\beta = 0$) of CRTI						-0.079**		-0.079**		-0.080**
S.e.						0.034		0.034		0.034
MFX P($\beta > 0$) of CRTI						0.132**		0.132**		0.132**
S.e.						0.058		0.058		0.058
MFX P($\beta = 0$) of CRTO						0.033		0.034		0.034
S.e.						0.047		0.047		0.047
MFX P($\beta > 0$) of CRTO						-0.055		-0.056		-0.056
S.e.						0.083		0.083		0.083
N	342		342		342		342		342	

Standard errors in parentheses. * $p < 0.10$, ** $p < 0.05$, *** $p < 0.01$

Table 4: Ordered probit regressions of social preferences individual estimates and marginal effects by using a dummy = 1 when the Left 2D:4D ratio (L2D:4D) is higher than the gender specific median.

	(1)		(2)		(3)		(4)		(5)	
	α	β	α	β	α	β	α	β	α	β
HL2D:4D (HL)	-0.006	-0.106	-0.002	-0.107	-0.007	-0.088	-0.146	0.033	0.208	-0.075
	(0.123)	(0.125)	(0.124)	(0.125)	(0.125)	(0.126)	(0.168)	(0.172)	(0.248)	(0.252)
Female (F)			0.376***	0.095	0.326**	0.060	0.172	0.191	0.318**	0.080
			(0.124)	(0.125)	(0.126)	(0.127)	(0.177)	(0.180)	(0.127)	(0.128)
CRT Imp (CRTI)					0.359**	0.339**	0.360**	0.340**	0.452**	0.423**
					(0.149)	(0.151)	(0.149)	(0.151)	(0.204)	(0.208)
CRT Others (CRTO)					0.261	-0.129	0.269	-0.138	0.750**	-0.611*
					(0.216)	(0.214)	(0.216)	(0.214)	(0.350)	(0.333)
HL × F							0.307	-0.257		
							(0.249)	(0.251)		
HL × CRTI									-0.194	-0.174
									(0.294)	(0.298)
HL × CRTO									-0.800*	0.757*
									(0.448)	(0.437)
MFX P(α=0) HL	0.001		0.000		0.001		0.000		0.003	
S.e.	0.020		0.020		0.021		0.021		0.021	
MFX P($\alpha > 0$) HL	-0.002		-0.001		-0.003		-0.000		-0.007	
S.e.	0.049		0.049		0.050		0.050		0.050	
MFX P(α=0) F			-0.062***		-0.054**		-0.055**		-0.055**	
S.e.			0.022		0.022		0.023		0.023	
MFX P($\alpha > 0$) F			0.148***		0.129***		0.129***		0.126**	
S.e.			0.049		0.050		0.050		0.050	
MFX P($\alpha = 0$) CRTI					-0.054**		-0.054**		-0.055**	
S.e.					0.022		0.022		0.022	
MFX P($\alpha > 0$) CRTI					0.141**		0.141**		0.140**	
S.e.					0.058		0.058		0.058	
MFX P($\alpha = 0$) CRTO					-0.050		-0.051		-0.070	
S.e.					0.046		0.046		0.051	
MFX P($\alpha > 0$) CRTO					0.104		0.107		0.139	
S.e.					0.085		0.085		0.088	
MFX P(β=0) HL		0.026		0.026		0.022		0.022		0.022
S.e.		0.031		0.031		0.031		0.032		0.031
MFX P($\beta > 0$) HL		-0.042		-0.042		-0.035		-0.035		-0.035
S.e.		0.049		0.049		0.050		0.050		0.050
MFX P(β=0) F				-0.023		-0.015		-0.016		-0.020
S.e.				0.031		0.032		0.032		0.032
MFX P($\beta > 0$) F				0.038		0.024		0.025		0.032
S.e.				0.049		0.050		0.050		0.050
MFX P($\beta = 0$) CRTI						-0.080**		-0.080**		-0.079**
S.e.						0.034		0.034		0.034
MFX P($\beta > 0$) CRTI						0.132**		0.133**		0.131**
S.e.						0.058		0.058		0.058
MFX P($\beta = 0$) CRTO						0.031		0.033		0.052
S.e.						0.048		0.048		0.043
MFX P($\beta > 0$) CRTO						-0.051		-0.054		-0.090
S.e.						0.083		0.083		0.083
N	342	342	342	342	342	342	342	342	342	342

Standard errors in parentheses

* $p < 0.10$, ** $p < 0.05$, *** $p < 0.01$

Table 5: Ordered probit regressions of social preferences individual estimates and marginal effects with L2D:4D in levels

	(1)		(2)		(3)		(4)		(5)	
	α	β	α	β	α	β	α	β	α	β
HL 2D:4D (HL)	-0.704	-1.312	-1.667	-1.612	-1.764	-1.592	-2.622	-1.521	1.173	-5.089
	(1.623)	(1.646)	(1.663)	(1.676)	(1.667)	(1.684)	(2.281)	(2.349)	(3.406)	(3.495)
Female (F)			0.393***	0.122	0.345***	0.088	-1.467	0.232	0.341***	0.106
			(0.127)	(0.127)	(0.129)	(0.129)	(3.284)	(3.316)	(0.129)	(0.130)
CRT Imp (CRTI)					0.360**	0.343**	0.359**	0.343**	2.460	-1.850
					(0.149)	(0.151)	(0.149)	(0.151)	(3.885)	(3.974)
CRT Others (CRTO)					0.275	-0.135	0.278	-0.136	12.973**	-15.726***
					(0.215)	(0.213)	(0.215)	(0.214)	(5.872)	(5.912)
HL × F							1.842	-0.147		
							(3.337)	(3.371)		
HL × CRT									-2.150	2.247
									(3.966)	(4.054)
HL × CRTO									-12.866**	15.839***
									(5.949)	(6.010)
MFX P(α=0) HL	0.112		0.271		0.291		0.286		0.314	
S.e.	0.257		0.271		0.276		0.273		0.286	
MFX P($\alpha > 0$) HL	-0.280		-0.661		-0.699		-0.694		-0.733	
S.e.	0.644		0.657		0.658		0.658		0.662	
MFX P(α=0) F			-0.064***		-0.057**		-0.056**		-0.058**	
S.e.			0.023		0.023		0.023		0.024	
MFX P($\alpha > 0$) F			0.155***		0.136***		0.135***		0.135***	
S.e.			0.050		0.050		0.050		0.051	
MFX P($\alpha = 0$) CRTI					-0.054**		-0.053**		-0.055**	
S.e.					0.022		0.021		0.022	
MFX P($\alpha > 0$) CRTI					0.141**		0.141**		0.138**	
S.e.					0.058		0.057		0.058	
MFX P($\alpha = 0$) CRTO					-0.052		-0.053		-0.069	
S.e.					0.046		0.046		0.050	
MFX P($\alpha > 0$) CRTO					0.109		0.111		0.137	
S.e.					0.085		0.085		0.087	
MFX P(β=0) HL		0.320		0.394		0.397		0.397		0.407
S.e.		0.401		0.408		0.419		0.419		0.427
MFX P(β >0) HL		-0.519		-0.637		-0.629		-0.629		-0.640
S.e.		0.650		0.660		0.663		0.664		0.669
MFX P(β=0) F				-0.030		-0.022		-0.022		-0.027
S.e.				0.031		0.033		0.033		0.033
MFX P($\beta > 0$) F				0.048		0.035		0.035		0.042
S.e.				0.050		0.051		0.051		0.051
MFX P($\beta = 0$) CRTI						-0.081**		-0.081**		-0.084**
S.e.						0.034		0.034		0.035
MFX P($\beta > 0$) CRTI						0.134**		0.134**		0.139**
S.e.						0.058		0.058		0.058
MFX P($\beta = 0$) CRTO						0.032		0.032		0.042
S.e.						0.047		0.048		0.046
MFX P($\beta > 0$) CRTO						-0.053		-0.053		-0.070
S.e.						0.083		0.083		0.083
N	341	341	341	341	341	341	341	341	341	341

Standard errors in parentheses

* $p < 0.10$, ** $p < 0.05$, *** $p < 0.01$

Table 6: Ordered probit regressions of social preferences individual estimates and marginal effects by using a dummy = 1 if L2D:4D is in the top-bottom tercile

	(1)		(2)		(3)		(4)		(5)	
	α	β	α	β	α	β	α	β	α	β
HL2D:4D (HL)	0.251*	-0.030	0.277**	-0.025	0.264**	-0.061	0.180	-0.126	0.565**	0.095
	(0.128)	(0.130)	(0.129)	(0.130)	(0.130)	(0.131)	(0.180)	(0.186)	(0.257)	(0.258)
Female (F)			0.393***	0.094	0.343***	0.058	0.232	-0.029	0.384***	0.065
			(0.125)	(0.125)	(0.127)	(0.127)	(0.210)	(0.215)	(0.130)	(0.130)
CRT Imp (CRTI)					0.345**	0.341**	0.356**	0.349**	0.499**	0.446*
					(0.149)	(0.151)	(0.150)	(0.152)	(0.246)	(0.250)
CRT Others (CRTO)					0.269	-0.145	0.255	-0.154	0.864**	0.070
					(0.215)	(0.213)	(0.216)	(0.214)	(0.339)	(0.330)
HL × F							0.175	0.134		
							(0.264)	(0.267)		
HL × CRTI									-0.262	-0.171
									(0.307)	(0.311)
HL × CRTO									-1.021**	-0.367
									(0.441)	(0.433)
MFX P(α=0) HL	-0.038**		-0.042**		-0.041**		-0.041**		-0.042**	
S.e.	0.019		0.019		0.020		0.020		0.020	
MFX P(α >0) HL	0.099**		0.109**		0.104**		0.104**		0.105**	
S.e.	0.050		0.050		0.051		0.051		0.051	
MFX P(α=0) F			-0.065***		-0.058**		-0.058**		-0.065***	
S.e.			0.023		0.023		0.023		0.024	
MFX P(α >0) F			0.155***		0.136***		0.137***		0.152***	
S.e.			0.049		0.050		0.050		0.051	
MFX P($\alpha = 0$) CRTI					-0.053**		-0.054**		-0.051**	
S.e.					0.022		0.022		0.022	
MFX P($\alpha > 0$) CRTI					0.136**		0.140**		0.130**	
S.e.					0.058		0.058		0.058	
MFX P($\alpha = 0$) CRTO					-0.052		-0.049		-0.038	
S.e.					0.046		0.046		0.045	
MFX P($\alpha > 0$) CRTO					0.107		0.101		0.081	
S.e.					0.085		0.085		0.086	
MFX P(β=0) HL		0.007		0.006		0.015		0.016		0.015
S.e.		0.032		0.032		0.033		0.034		0.034
MFX P(β >0) HL		-0.012		-0.010		-0.024		-0.025		-0.024
S.e.		0.052		0.052		0.052		0.052		0.052
MFX P(β=0) F				-0.023		-0.014		-0.014		-0.016
S.e.				0.031		0.032		0.032		0.033
MFX P(β >0) F				0.037		0.023		0.023		0.026
S.e.				0.049		0.050		0.050		0.051
MFX P($\beta = 0$) CRTI						-0.080**		-0.082**		-0.079**
S.e.						0.034		0.034		0.034
MFX P($\beta > 0$) CRTI						0.133**		0.137**		0.131**
S.e.						0.058		0.058		0.058
MFX P($\beta = 0$) CRTO						0.034		0.036		0.039
S.e.						0.047		0.047		0.046
MFX P($\beta > 0$) CRTO						-0.057		-0.060		-0.065
S.e.						0.083		0.083		0.083
N	342	342	342	342	342	342	342	342	342	342

Standard errors in parentheses

* $p < 0.10$, ** $p < 0.05$, *** $p < 0.01$

Table 7: Subjects' consistency in risky choices using R2D:4D in level

	(1)	(2)	(3)	(4)	(5)
HR2D:4D (HR)	0.705	0.938*	0.970*	0.448	-0.283
	(0.480)	(0.500)	(0.502)	(0.696)	(1.050)
Female (F)		-0.074*	-0.041	-1.084	-0.040
		(0.039)	(0.040)	(0.963)	(0.039)
CRT Imp. (CRTI)			-0.167***	-0.165***	-2.019*
			(0.039)	(0.040)	(1.167)
CRT Other (CRTO)			-0.156***	-0.152***	-0.398
			(0.053)	(0.052)	(1.477)
HR × F				1.066	
				(0.979)	
HR × CRTI					1.899
					(1.197)
HR × CRTO					0.254
					(1.517)
Project 1	0.064	0.067	0.063	0.060	0.061
	(0.044)	(0.045)	(0.045)	(0.045)	(0.045)
Constant	0.084	-0.109	-0.024	0.481	1.194
	(0.470)	(0.485)	(0.484)	(0.674)	(1.016)
MFX of F				-0.042	
S.e.				0.040	
MFX of CRTI					-0.162***
S.e.					0.039
MFX of CRTO					-0.150***
S.e.					0.053
MFX of HR				0.958*	0.913*
S.e.				0.499	0.496
N	497	497	497	497	497

Robust standard errors in parentheses. * $p < 0.10$, ** $p < 0.05$, *** $p < 0.01$

Table 8: Subjects' relative frequency of risky choices for consistent subjects using R2D:4D in level

	(1)	(2)	(3)	(4)	(5)
HR2D:4D (HR)	-0.049	0.159	0.165	0.082	-0.129
	(0.204)	(0.217)	(0.217)	(0.272)	(0.460)
Female (F)		-0.061***	-0.059***	-0.230	-0.059***
		(0.018)	(0.019)	(0.433)	(0.019)
CRT Imp. (CRTI)			-0.007	-0.007	-0.245
			(0.020)	(0.020)	(0.515)
CRT Other (CRTO)			0.006	0.007	-0.697
			(0.025)	(0.025)	(0.575)
HR × F				0.174	
				(0.440)	
HR × CRTI					0.245
					(0.527)
HR × CRTO					0.722
					(0.588)
Project 1	-0.063***	-0.059***	-0.059***	-0.059***	-0.058***
	(0.019)	(0.019)	(0.019)	(0.019)	(0.019)
Constant	0.503**	0.327	0.323	0.404	0.608
	(0.199)	(0.210)	(0.211)	(0.263)	(0.448)
MFX of F				-0.060***	
S.e.				0.019	
MFX of CRTI					-0.005
S.e.					0.020
MFX of CRTO					0.008
S.e.					0.025
MFX of HR				0.165	0.158
S.e.				0.218	0.215
N	390	390	390	390	390

Robust standard errors in parentheses. * $p < 0.10$, ** $p < 0.05$, *** $p < 0.01$

Table 9: Subjects' consistency in risky choices by using a dummy = 1 if R2D:4D in top-bottom tercile

	(1)	(2)	(3)	(4)	(5)
HR2D:4D (HR)	-0.010	-0.003	0.002	0.024	0.009
	(0.039)	(0.039)	(0.039)	(0.050)	(0.056)
Female (F)		-0.057	-0.024	0.011	-0.028
		(0.037)	(0.039)	(0.065)	(0.039)
CRT Imp. (CRTI)			-0.166***	-0.168***	-0.137**
			(0.039)	(0.039)	(0.063)
CRT Other (CRTO)			-0.154***	-0.154***	-0.207**
			(0.052)	(0.052)	(0.089)
HR × F				-0.051	
				(0.078)	
HR × CRTI					-0.040
					(0.078)
HR × CRTO					0.084
					(0.109)
Project 1	0.070	0.074*	0.070	0.070	0.077*
	(0.044)	(0.045)	(0.045)	(0.045)	(0.044)
Constant	0.779***	0.801***	0.912***	0.899***	0.907***
	(0.033)	(0.036)	(0.037)	(0.042)	(0.045)
MFX of F				-0.023	
S.e.				0.039	
MFX of CRTI					-0.164***
S.e.					0.039
MFX of CRTO					-0.150***
S.e.					0.052
MFX of HR				0.000	0.000
S.e.				0.039	0.039
N	497	497	497	497	497

Robust standard errors in parentheses. * $p < 0.10$, ** $p < 0.05$, *** $p < 0.01$

Table 10: Subjects' relative frequency of risky choices for consistent subjects by using a dummy = 1 if R2D:4D in top-bottom tercile

	(1)	(2)	(3)	(4)	(5)
HR2D:4D (HR)	0.020	0.026	0.026	0.032	0.019
	(0.017)	(0.017)	(0.017)	(0.021)	(0.032)
Female (F)		-0.060***	-0.058***	-0.049*	-0.058***
		(0.017)	(0.018)	(0.029)	(0.018)
CRT Imp. (CRTI)			-0.008	-0.008	-0.013
			(0.020)	(0.020)	(0.031)
CRT Other (CRTO)			0.006	0.006	-0.003
			(0.025)	(0.026)	(0.042)
HR × F				-0.013	
				(0.035)	
HR × CRTI					0.009
					(0.040)
HR × CRTO					0.014
					(0.052)
Project 1	-0.063***	-0.056***	-0.056***	-0.056***	-0.056***
	(0.019)	(0.019)	(0.019)	(0.019)	(0.019)
Constant	0.442***	0.465***	0.467***	0.464***	0.471***
	(0.014)	(0.015)	(0.019)	(0.019)	(0.024)
MFX of F				-0.058***	
S.e.				0.018	
MFX of CRTI					-0.007
S.e.					0.020
MFX of CRTO					0.006
S.e.					0.026
MFX of HR				0.025	0.026
S.e.				0.017	0.017
N	390	390	390	390	390

Robust standard errors in parentheses. * $p < 0.10$, ** $p < 0.05$, *** $p < 0.01$

Table 11: Subjects' consistency in risky choices using L2D:4D in level

	(1)	(2)	(3)	(4)	(5)
HL2D:4D (HL)	0.424	0.633	0.703	0.818	0.820
	(0.556)	(0.566)	(0.567)	(0.765)	(1.117)
Female (F)		-0.068*	-0.035	0.197	-0.036
		(0.038)	(0.039)	(1.117)	(0.039)
CRT Imp. (CRTI)			-0.169***	-0.169***	-0.134
			(0.039)	(0.039)	(1.308)
CRT Other (CRTO)			-0.155***	-0.156***	0.298
			(0.052)	(0.052)	(1.657)
HL × F				-0.237	
				(1.133)	
HL × CRTI					-0.036
					(1.331)
HL × CRTO					-0.462
					(1.689)
Project 1	0.069	0.072	0.068	0.068	0.067
	(0.044)	(0.045)	(0.045)	(0.045)	(0.045)
Constant	0.356	0.182	0.230	0.119	0.117
	(0.547)	(0.554)	(0.555)	(0.749)	(1.094)
MFX of F				-0.035	
S.e.				0.039	
MFX of CRTI					-0.169***
S.e.					0.039
MFX of CRTO					-0.156***
S.e.					0.052
MFX of HL				0.705	0.709
S.e.				0.567	0.569
N	496	496	496	496	496

Standard errors in parentheses

* $p < 0.10$, ** $p < 0.05$, *** $p < 0.01$

Table 12: Subjects' relative frequency of risky choices for consistent subjects using L2D:4D in level

	(1)	(2)	(3)	(4)	(5)
HL2D:4D (HL)	-0.117	0.072	0.079	0.038	0.529
	(0.248)	(0.256)	(0.255)	(0.315)	(0.465)
Female (F)		-0.060^{***}	-0.058^{***}	-0.145	-0.058^{***}
		(0.018)	(0.018)	(0.515)	(0.018)
CRT Imp. (CRTI)			-0.007	-0.007	0.626
			(0.020)	(0.020)	(0.562)
CRT Other (CRTO)			0.007	0.007	0.380
			(0.025)	(0.025)	(0.674)
HL × F				0.088	
				(0.522)	
HL × CRTI					-0.646
					(0.572)
HL × CRTO					-0.383
					(0.686)
Project 1	-0.063^{***}	-0.057^{***}	-0.057^{***}	-0.057^{***}	-0.057^{***}
	(0.019)	(0.019)	(0.019)	(0.019)	(0.019)
Constant	0.570^{**}	0.410	0.406	0.446	-0.034
	(0.243)	(0.249)	(0.250)	(0.308)	(0.457)
MFX of F				-0.058^{***}	
S.e.				0.019	
MFX of CRTI					-0.009
S.e.					0.020
MFX of CRTO					0.004
S.e.					0.025
MFX of HL				0.080	0.066
S.e.				0.257	0.254
N	389	389	389	389	389

Standard errors in parentheses

* $p < 0.10$, ** $p < 0.05$, *** $p < 0.01$

Table 13: Subjects' consistency in risky choices by using a dummy = 1 when the L2D:4D higher than the gender specific median

	(1)	(2)	(3)	(4)	(5)
HL2D:4D (HL)	0.040	0.040	0.045	0.050	-0.012
	(0.037)	(0.037)	(0.037)	(0.048)	(0.055)
Female (F)		-0.057	-0.024	-0.019	-0.021
		(0.037)	(0.038)	(0.055)	(0.038)
CRT Imp. (CRTI)			-0.167***	-0.168***	-0.218***
			(0.039)	(0.039)	(0.054)
CRT Other (CRTO)			-0.157***	-0.157***	-0.139*
			(0.052)	(0.052)	(0.071)
HL × F				-0.010	
				(0.073)	
HL ×CRTI					0.103
					(0.074)
HL ×CRTO					-0.030
					(0.103)
Project 1	0.068	0.071	0.067	0.067	0.064
	(0.044)	(0.045)	(0.045)	(0.045)	(0.045)
Constant	0.752***	0.779***	0.893***	0.891***	0.919***
	(0.028)	(0.032)	(0.034)	(0.038)	(0.037)
MFX of F				-0.024	
S.e.				0.039	
MFX of CRTI					-0.167
S.e.					0.039
MFX of CRTO					-0.154
S.e.					0.052
MFX wrt HL				0.045	0.045
S.e.				0.037	0.036
N	497	497	497	497	497

Standard errors in parentheses

* $p < 0.10$, ** $p < 0.05$, *** $p < 0.01$

Table 14: Subjects' relative frequency of risky choices for consistent subjects by using a dummy = 1 when the L2D:4D higher than the gender specific median

	(1)	(2)	(3)	(4)	(5)
HL2D:4D (HL)	0.012	0.012	0.012	0.029	0.010
	(0.017)	(0.017)	(0.016)	(0.020)	(0.031)
Female (F)		-0.058***	-0.056***	-0.037	-0.056***
		(0.017)	(0.018)	(0.024)	(0.018)
CRT Imp. (CRTI)			-0.007	-0.009	-0.008
			(0.020)	(0.020)	(0.029)
CRT Other (CRTO)			0.006	0.005	0.003
			(0.025)	(0.025)	(0.036)
HL × F				-0.037	
				(0.034)	
HL ×CRTI					0.001
					(0.038)
HL ×CRTO					0.007
					(0.050)
Project 1	-0.064***	-0.058***	-0.058***	-0.058***	-0.058***
	(0.019)	(0.019)	(0.019)	(0.019)	(0.019)
Constant	0.450***	0.475***	0.477***	0.469***	0.478***
	(0.012)	(0.013)	(0.018)	(0.019)	(0.023)
MFX of F				-0.055	
S.e.				0.018	
MFX of CRTI					-0.007
S.e.					0.020
MFX of CRTO					0.006
S.e.					0.025
MFX of HL				0.012	0.012
S.e.				0.017	0.017
N	390	390	390	390	390

Standard errors in parentheses

* $p < 0.10$, ** $p < 0.05$, *** $p < 0.01$

Table 15: Subjects' consistency in risky choices by using a dummy = 1 if L2D:4D in top-bottom tercile

	(1)	(2)	(3)	(4)	(5)
HL2D:4D (HL)	-0.008	-0.009	0.001	-0.021	-0.050
	(0.039)	(0.038)	(0.038)	(0.050)	(0.053)
Female (F)		-0.057	-0.024	-0.054	-0.020
		(0.037)	(0.038)	(0.065)	(0.039)
CRT Imp. (CRTI)			-0.166***	-0.161***	-0.213***
			(0.039)	(0.040)	(0.059)
CRT Other (CRTO)			-0.154***	-0.153***	-0.176**
			(0.052)	(0.052)	(0.084)
HL × F				0.044	
				(0.078)	
HL × CRTI					0.074
					(0.076)
HL × CRTO					0.037
					(0.107)
Project 1	0.071	0.075*	0.070	0.071	0.070
	(0.044)	(0.044)	(0.044)	(0.044)	(0.045)
Constant	0.777***	0.805***	0.913***	0.925***	0.942***
	(0.033)	(0.035)	(0.036)	(0.040)	(0.035)
MFX of F				-0.025	
S.e.				0.039	
MFX of CRTI					-0.164***
S.e.					0.040
MFX of CRTO					-0.151***
S.e.					0.053
MFX of HL				0.000	0.002
S.e.				0.038	0.039
N	497	497	497	497	497

Standard errors in parentheses

* $p < 0.10$, ** $p < 0.05$, *** $p < 0.01$

Table 16: Subjects' relative frequency of risky choices for consistent subjects by using a dummy = 1 if L2D:4D in top-bottom tercile

	(1)	(2)	(3)	(4)	(5)
HL2D:4D (HL)	-0.006	-0.005	-0.005	0.003	0.039
	(0.017)	(0.017)	(0.017)	(0.021)	(0.032)
Female (F)		-0.058***	-0.056***	-0.044	-0.059***
		(0.017)	(0.018)	(0.028)	(0.018)
CRT Imp. (CRTI)			-0.006	-0.007	0.035
			(0.020)	(0.020)	(0.031)
CRT Other (CRTO)			0.007	0.007	0.034
			(0.025)	(0.025)	(0.041)
HL × F				-0.018	
				(0.035)	
HL × CRTI					-0.064
					(0.040)
HL × CRTO					-0.045
					(0.052)
Project 1	-0.064***	-0.058***	-0.057***	-0.058***	-0.058***
	(0.019)	(0.019)	(0.019)	(0.019)	(0.019)
Constant	0.459***	0.484***	0.486***	0.481***	0.461***
	(0.013)	(0.014)	(0.019)	(0.019)	(0.024)
MFX of F				-0.056***	
S.e.				0.018	
MFX of CRTI					-0.008
S.e.					0.020
MFX of CRTO					0.004
S.e.					0.026
MFX of HL				-0.006	-0.009
S.e.				0.017	0.017
N	390	390	390	390	390

Standard errors in parentheses

* $p < 0.10$, ** $p < 0.05$, *** $p < 0.01$
